# Specific pools of endogenous peptides are present in gametophore, protonema, and protoplast cells of the moss *Physcomitrella patens*

**DOI:** 10.1186/s12870-015-0468-7

**Published:** 2015-03-15

**Authors:** Igor A Fesenko, Georgij P Arapidi, Alexander Yu Skripnikov, Dmitry G Alexeev, Elena S Kostryukova, Alexander I Manolov, Ilya A Altukhov, Regina A Khazigaleeva, Anna V Seredina, Sergey I Kovalchuk, Rustam H Ziganshin, Viktor G Zgoda, Svetlana E Novikova, Tatiana A Semashko, Darya K Slizhikova, Vasilij V Ptushenko, Alexey Y Gorbachev, Vadim M Govorun, Vadim T Ivanov

**Affiliations:** Department of Proteomics, Shemyakin-Ovchinnikov Institute of Bioorganic Chemistry of the Russian Academy of Sciences, 16/10, Miklukho-Maklaya, GSP-7, Moscow, 117997 Russian Federation; Research Institute of Physical-Chemical Medicine, Federal Medical & Biological Agency, 1a, Malaya Pirogovskaya, Moscow, 119992 Russian Federation; Moscow Institute of Physics and Technology, 9 Institutskiy per., Dolgoprudny, Moscow Region, 141700 Russian Federation; Institute of Biomedical Chemistry RAMS im. V.N. Orehovicha, 10, Pogodinskaya Street, Moscow, 119121 Russian Federation; A. N. Belozersky Institute of Physico-Chemical Biology, M.V. Lomonosov Moscow State University, Leninskye Gory, House 1, Building 40, Moscow, 119992 Russian Federation; Biology Department, Lomonosov Moscow State University, Moscow, 199234 Russian Federation

**Keywords:** Endogenous peptides, LC-MS/MS, Physcomitrella patens, Proteome, Transcriptome profiling

## Abstract

**Background:**

Protein degradation is a basic cell process that operates in general protein turnover or to produce bioactive peptides. However, very little is known about the qualitative and quantitative composition of a plant cell peptidome, the actual result of this degradation. In this study we comprehensively analyzed a plant cell peptidome and systematically analyzed the peptide generation process.

**Results:**

We thoroughly analyzed native peptide pools of *Physcomitrella patens* moss in two developmental stages as well as in protoplasts. Peptidomic analysis was supplemented by transcriptional profiling and quantitative analysis of precursor proteins. In total, over 20,000 unique endogenous peptides, ranging in size from 5 to 78 amino acid residues, were identified. We showed that in both the protonema and protoplast states, plastid proteins served as the main source of peptides and that their major fraction formed outside of chloroplasts. However, in general, the composition of peptide pools was very different between these cell types. In gametophores, stress-related proteins, e.g., late embryogenesis abundant proteins, were among the most productive precursors. The Driselase-mediated protonema conversion to protoplasts led to a peptide generation “burst”, with a several-fold increase in the number of components in the latter. Degradation of plastid proteins in protoplasts was accompanied by suppression of photosynthetic activity.

**Conclusion:**

We suggest that peptide pools in plant cells are not merely a product of waste protein degradation, but may serve as important functional components for plant metabolism. We assume that the peptide “burst” is a form of biotic stress response that might produce peptides with antimicrobial activity from originally functional proteins. Potential functions of peptides in different developmental stages are discussed.

**Electronic supplementary material:**

The online version of this article (doi:10.1186/s12870-015-0468-7) contains supplementary material, which is available to authorized users.

## Background

Peptides are well known to be key regulators of many animal physiological processes, including defense reactions and hormonal, neurohumoral, and signaling functions. In recent years, a number of small peptides with similar activities have been also discovered in land plants [1-4]. As in animals, peptide signals regulating plant growth and development act as ligands of receptor-like kinases [5]. Over 400 homologs of receptor-like kinases and more than 1000 genes predicted to encode precursors of secreted peptides are found in the genome of *Arabidopsis thaliana* [6,7]. Thus, the currently known regulatory plant peptides very likely constitute just a tiny portion of the total number of secreted peptides really involved in the control of physiological processes [7]. Bioactive peptides are assumed to be primarily translated as inactive precursor proteins that are cleaved by various proteases to produce matured bioactive factors. In recent years, a new source of bioactive peptides has been found. Small open reading frames (ORFs) can be directly translated into peptides that play essential roles in eukaryotes [8-11]. In addition, degradation of originally functional proteins can also contribute to functional peptidomes in eukaryotic organisms [2,12-17]. Examples of such peptides in plants are inseptin, which is a fragment of chloroplast ATP synthase from cowpea (*Vigna unguiculata*) [18], and the GmSubPep and GmPep914 peptides produced from soy (*Glycine max*) subtilisin-like protease [19,20]. Still, little is known about the generation of the proteolytic degradome in plant cells and tissues or its physiological role.

The moss *Physcomitrella patens* is a promising model organism in plant biology [21-23]. Mosses are descendants of early divergent embryophyte lines and therefore occupy an ideal phylogenetic position for reconstructing the evolutionary history of terrestrial plants and understanding the changes that accompanied the emergence of land plants. Furthermore, *P. patens* exhibits the highest rate of homologous recombination among land plants, giving it the unique ability to be genetically manipulated using targeted gene replacements. In addition to its nuclear genome [24], numerous studies of the proteome [25-31], transcriptome [32-36], and metabolome [37] of *P. patens* have been published.

The gametophyte, the haploid generation that prevails in the moss life cycle, goes through two stages of development. In the first stage, called the protonema, the gametophyte is a net of filaments that develops in a wet environment. Protonema cells differentiate into buds that give rise to the leafy adult stage, termed the gametophore. Gametophores grow as three-dimensional leafy shoots on which the reproductive organs, antheridia and archegonia, form under suitable environmental conditions. Protoplasts prepared from protonema filaments are of particular interest because, during the first hours of regeneration, they are reprogrammed into protonemal apical stem cells without forming a callus. Protoplasts are useful for studies of stress because the isolation of protoplasts from cell walls appears to have similar effects to plasmolysis induced by drought or salinity stress [35]. We previously described the significant change in the proteome of *P. patens* protonema cells that occurs during protoplast isolation [38].

Peptides formed by degradation of functionally active proteins can represent a significant fraction of the cell peptidome, but this fraction is poorly understood in plant cells. The aim of this work was to identify the pools of native peptides, elucidate their patterns of formation, and evaluate the effects of stress factors on the peptidome. We comprehensively analyzed the peptidomes of protoplasts, protonemata, and gametophores of moss *P. patens* cells and performed transcriptional profiling and quantitative proteomic analysis of precursor proteins. Significant differences between the peptidomes of the three cell types were found. We did not observe direct proportionality between intact protein concentrations and their corresponding native peptide fragments; the intensity of degradation and proteolysis patterns depended, rather, on the moss cell form. This fact suggests that differentially regulated mechanisms of protein degradation are involved at different growth stages and that different peptides may be important for different cell forms. Under stress conditions, we found significant differences in the peptidome of moss protoplasts compared with protonemata and gametophores. An increase in the number of chloroplast protein peptides was accompanied by suppression of photosynthetic activity. We suggested that peptide pools generated by protein turnover and degradation have a significant potential for biological activity. We identified 81 peptides in protoplasts with probable antimicrobial activity. Finally, we suggested a scheme of processes leading to and affecting peptidome formation in protoplast cells.

## Methods

### *Physcomitrella patens* protonema and gametophore growth conditions

The protonemata of the moss *P. patens* subsp. *patens* Gransden 2004 were grown on Knop medium with 500 mg/L ammonium tartrate with 1.5%agar (Helicon, Moscow, Russian Federation) in a Sanyo Plant Growth Incubator MLR-352H (Panasonic, Osaka, Japan) with a photon flux of 61 μmol/m^2^•s during a 16-hour photoperiod at 24°C. For transcriptomic and peptidomic analyses, we used 5-day-old protonema tissue. The moss gametophores were grown on Knop medium in 9-cm Petri dishes in the same incubator with a 16-hour photoperiod at 24°C and 61 μmol/m^2^•s. We used 8-week-old gametophores for analyses.

### Protoplast preparation and driselase treatment

Five-day-old protonema filaments were harvested with a spatula from the agar surface, and 1 g well-drained protonema tissue was placed in 14 mL 0.5% (w/v) Driselase (Sigma-Aldrich, St. Louis, MO, USA) solution in 0.48 М mannitol and incubated for 60 min with constant shaking in darkness. Then, the suspension was filtered through 100 μm steel mesh (Sigma-Aldrich), and the protoplasts obtained were incubated in Driselase solution for 15 minutes more. The protoplasts were then precipitated by centrifugation in 50-mL plastic tubes using a swinging bucket rotor at 100 × *g* for 5 min. Next, protoplasts were washed twice with 0.48 М mannitol with centrifugation under the same conditions and sedimented again. The supernatant was removed and the protoplast pellet was frozen in liquid nitrogen for peptide extraction or RNA isolation. The number of protoplasts was measured with a hematocytometer.

The treatment of protonemata with 0.025% w/v and 0.0025% w/v Driselase solution followed a similar protocol. As a control, we also incubated protonema tissue in 0.48 М mannitol. After 1-h incubation, the protonema tissue was washed and peptides extracted.

### Isolation of chloroplasts from moss protoplasts

Chloroplasts were isolated from protoplasts as previously described [27]. In short, protoplasts were resuspended in buffer A (50 mM HEPES-KOH, pH 7.5, 330 mM sorbitol, 2 mM EDTA, and 0.4 mM phenylmethylsulfonyl fluoride) and filtered through a double layer of Miracloth (Calbiochem Behring, La Jolla, CA, USA). Protoplast disintegration was evaluated with a light microscope. The filtrate was then centrifuged at 1200 × *g* for 3 min in 50-mL plastic tubes using a bucket rotor. The pellet was resuspended in a small volume of buffer A and fractionated by centrifugation in a bucket rotor at 3800 × *g* for 10 min in a 10%-40%-85% Percoll (Sigma-Aldrich) stepwise gradient in 15-mL plastic tubes. Intact chloroplasts between the 40% and 85% Percoll layers were gathered, washed with buffer A, and centrifuged at 1200 × *g* for 3 min in 15-mL plastic tubes (Falcon) in a bucket rotor. The resulting chloroplast pellet was used for native peptide extraction.

### Peptide extraction

Endogenous peptides were extracted from moss tissue, protoplasts, and intact chloroplasts as previously described with some modification [38]. To minimize artifacts during peptide extraction, we used an acid extraction buffer with a mixture of plant protease inhibitors, and all steps were performed on ice. For peptide extraction from moss tissues and protoplasts, the extraction buffer was 1 М acetic acid in 10% acetonitrile and 10 μL/mL of Protease Inhibitor Cocktail (Sigma-Aldrich). Protoplasts and intact chloroplasts were disrupted directly in the extraction buffer with a Ultra-Turrax T10 basic homogenizer (IKA, Staufen, Germany) using a S10N10G nozzle at a rotation speed of 3000 rpm at 4°C. For peptide extraction, protonemata were harvested from the surface of the agar medium and gametophores were excised 1 mm above the agar surface. The tissue was then placed into a porcelain mortar pre-cooled to −70°C, where it was immediately frozen with liquid nitrogen and ground to fine dust with a pestle pre-cooled to −70°C. The ground material was placed into cooled extraction buffer containing proteinase inhibitors and homogenized using a Dismembrator S ball mill (Sartorius, Göttingen, Germany) at 2600 rpm for 2 min with a mix of glass balls of 0.1, 0.3, and 1 mm diameter (Sartorius). The suspension was centrifuged at 11,000 × *g* for 10 min at 4°C. The supernatant was then transferred to a clean test tube and centrifuged again at 11,000 × *g* for 10 min at 4°C, after which the pellet was discarded.

Samples were immediately placed into a gel filtration column to extract and fractionate the peptides. Gel filtration was carried out on a 2.5 cm × 30 cm column filled with Sephadex G-25 superfine in 0.1 M acetic acid. The elution was with 0.1 M acetic acid at a flow rate of 1 mL/min. Proteins and peptides were detected on an LKB Bromma 2518 Uvicord SD device (LKB, Vienna, Austria) at a wavelength of 280 nm. The fractions containing peptides were lyophilized and resuspended in 5% acetonitrile-0.1% trifluoroacetic acid. Before recording the mass spectra, samples were desalted on reversed-phase C18 microcolumns, which were prepared in 200 μL tips for an automatic pipette with two layers of Empore™ extraction disk reversed-phase C18 membrane (Supelco, Bellefonte, PA, USA) 1.6 mm in diameter, as previously described [39]. The desalted peptide preparations were concentrated on a SpeedVac Concentrator vacuum centrifugal evaporator (Savant, Waltham, MA, USA) to a volume of 5 μL and diluted with 3% acetonitrile in 0.1% trifluoroacetic acid to 20 μL.

### Protein extraction

Proteins were extracted using a modified phenol extraction procedure [40]. Plant tissue was ground to fine powder in liquid nitrogen, and three volumes of ice-cold extraction buffer (500 m Tris–HCl, pH 8.0, 50 mM EDTA, 700 mM sucrose, 100 mM КCl, 1 mM phenylmethylsulfonyl fluoride, 2% 2-mercaptoethanol, 1% Triton X*-*100) were added, followed by 10 min incubation on ice. An equal volume of ice-cold Tris–HCl (pH 8.0)-saturated phenol was added, and the mixture was vortexed and incubated for 10 min with shaking. After centrifugation (10 min, 5500 × *g*, 4°C), the phenol phase was collected and re-extracted twice with extraction buffer. Proteins were precipitated from the final phenol phase with three volumes of ice-cold 0.1 M ammonium acetate in methanol overnight at −20°C. The pellets were rinsed with ice-cold 0.1 M ammonium acetate in methanol three times and with ice-cold acetone containing 13 mM dithiothreitol once and then dried. Pellets were solubilized in a sample buffer (8 М urea, 2 М thio urea, 17% solution of 30% CHAPS (3-[(3-cholamidopropyl) dimethylammonio]-1-propanesulfonate) and 10% NP40 (octylphenoxypolyethoxyethanol)). Protein concentration in the samples was determined according to Bradford procedure using the Quick Start Bradford protein assay (Bio-Rad, Hercules, CA USA); bovine serum albumin was used to prepare standard solutions.

### Mass-spectrometry analysis

Analysis was performed on a TripleTOF 5600+ mass-spectrometer with NanoSpray III ion source (ABSciex, Framingham, MA 01701, USA) coupled with a NanoLC Ultra 2D+ nano-HPLC system (Eksigent, Dublin, CA, USA). The HPLC system was configured in a trap–elute mode. For sample loading buffer and buffer A, a mix of 98.9% water, 1% methanol (v/v), and 0.1% formic acid (v/v) was used. Buffer B was 99.9% acetonitrile and 0.1% formic acid (v/v). Samples were loaded on a trap column Chrom XP C18 (3 μm, 120 Å, 350 μm × 0.5 mm; Eksigent) at a flow rate of 3 μL/min for 10 min and eluted through the separation column 3C18-CL-120 (3 μm, 120 Å, 75 μm × 150 mm; Eksigent) at a flow rate of 300 nL/min. The gradient was from 5% to 40% buffer B over 120 min. The column and precolumn were regenerated between runs by a wash with 95% buffer B for 7 min and equilibrated with 5% buffer B for 25 min. To thoroughly wash the column and precolumn between different samples and to prevent possible crosstalk, a 45-min blank run consisting of 5 × 5 min waves (5%, 95%, 95%, and 5% B) was performed, followed by column equilibration for 10 min with 5% B.

An information-dependent mass-spectrometer (MS) experiment included one survey MS1 scan followed by 50 dependent MS2 scans. MS1 acquisition parameters were 300–1250 m/z mass range for analysis and subsequent ion selection for MS2 analysis and 250 ms signal accumulation time. Ions for MS2 analysis were selected on the basis of intensity with a threshold of 400 cps and a charge state from 2 to 5. MS2 acquisition parameters were: resolution of quadrupole set to UNIT (0.7 Da), measurement mass range 200–1800 m/z, optimization of ion beam focus to obtain maximal sensitivity, and signal accumulation time of 50 ms for each parent ion. Collision activated dissociation was performed with nitrogen gas with collision energy ramping from 25 to 55 V within the 50-ms signal accumulation time. Analyzed parent ions were sent to a dynamic exclusion list for 15 sec to get an MS2 spectrum at the chromatographic peak apex (minimum peak width throughout the gradient was about 30 s).

An LTQ Orbitrap Velos system was equipped with an Agilent HPLC System 1100 Series (Agilent Technologies, Santa Clara, CA, USA) and a nanoelectrospray ion source (Thermo Scientific, Waltham, MA, USA). The peptide separation was carried out on an RP-HPLC column Zorbax 300SB-C18 (Agilent Technologies, Santa Clara, CA 95051, USA) (3.5 μm × 75 μm × 150 mm) using a linear gradient from 95% solvent A (100% water, 0.1% formic acid) and 5% solvent B (20% water, 80% acetonitrile, 0.1% formic acid) to 40% solvent A and 60% solvent B over 85 minutes at a flow rate of 300 nL/min.

Mass spectra were acquired in positive ion mode. Data were acquired in the Orbitrap analyzer with a resolution of 30,000 (m/z 400) for MS and 7,500 (m/z 400) for MS/MS scans. A survey MS scan was followed by acquisition of MS/MS spectra of the five most abundant precursors. For peptide fragmentation, high-energy collisional dissociation (HCD) was used; the signal threshold was set to 5,000 for an isolation window of 2 Th and the first mass of an HCD spectrum was set to 100 m/z. The collision energy was set to 35 eV. Fragmented precursors were dynamically excluded from targeting for 60 s. Singly charged ions and ions with a non-defined charge state were excluded from triggering MS/MS scans.

### Relative protein quantification

Protein concentrations were evaluated by label-free MS1 intensity-based quantification with the use of the Progenesis LC-MS (Nonlinear Dynamics, Durham, NC, USA) software package, which estimated correlation of tryptic peptidogenicity and protein expression levels. Raw data files (.wiff format) were converted into .mzML files using AB SCIEX MS Data Converter (version 1.3, ABSciex) and loaded into the Progenesis LC-MS. Progenesis LC-MS generated mascot generic files (.mgf format) that were searched using Mascot version 2.4.1 (Matrix Science, Boston, MA 02110, USA) against the UniProt sequence database (UniProt Consortium, (ftp.uniprot.org/pub/databases/uniprot/current_release/knowledgebase/complete, downloaded April 19, 2010) filtered by *P. patens* proteins (35,414 amino acid sequences). The Mascot search was performed with the following parameters: tryptic-specific peptides; maximum of one missed cleavage; peptide charge state limited to 1+, 2+, and 3+; precursor mass tolerance 20 ppm; MS/MS mass tolerance 50 ppm; variable modifications caused by oxidation (M) and carbamidomethylation (C). Using decoy (reversed) databases, false discovery rates (FDRs) were calculated, and the ion score cut-off was set to an FDR less than 5%.

Two-sided unpaired Student’s t-test (R version 3.0.2; R Foundation, Vienna, Austria) was conducted to evaluate the validity of the quantification results. Log (base 2) fold changes between different conditions were calculated for median values.

### Peptide analysis by mass spectrometry and data integration

Moss native peptide identifications were performed on the basis of a single LC-MS run for each sample. The .wiff data files were analyzed with the ProteinPilot software 4.5 revision 1656 (ABSciex) using the search algorithm Paragon 4.5.0.0 revision 1654 and the default parameter set for protein identification with the following adjustments: uniref100_Physco_35213 protein sequence database no Cys alkylation, no digestion, TripleTOF5600 equipment, organism type not specified, search effort – thorough ID, detection protein threshold – unused protein score 0.05. Spectrum grouping was performed with default parameters using the ProGroup algorithm embedded in ProteinPilot. Peptide identification FDR statistical analysis was performed using the ProteomicS Performance Evaluation Pipeline Software (PSPEP) algorithm also embedded in the ProteinPilot software. Peptides with probability over 95% were selected for analysis. Additionally, spectra acquired with TripleTOF 5600+ and LTQ Orbitrap Velos were searched with Mascot Version: 2.2.07 (Matrix Science), using the following parameters: precursor mass tolerance 20 ppm, MS/MS mass tolerance 50 ppm, no fixed modifications. Peptides with Mascot scores above the threshold were selected for analysis.

Peptide identification data was integrated in an *ad hoc* SQL database based on protein accessions. The number of peptides per protein was calculated as a sum of unique peptides found by both search algorithms. The functional analysis of precursor proteins was performed with the Database for Annotation, Visualization and Integrated Discovery (DAVID) (http://david.abcc.ncifcrf.gov/). The following parameters were used: count threshold 2, EASE threshold 0.01. Parameters of cluster analysis were as follows: similarity term overlap 10, similarity threshold 0.50, initial and final group membership 3, multiple linkage threshold 0.5, and enrichment thresholds 0.01.

### Antimicrobial peptide potential

The native peptide theoretical antimicrobial potential was assessed on the basis of sequence with special AMPA software (http://tcoffee.crg.cat/apps/ampa/do) [41]. All identified native peptides longer than six amino acids were searched for any internal part with high antimicrobial potential. For the AMPA analysis, the recommended parameters were used: threshold value of 0.225 and window size of seven amino acids. Only antimicrobial peptides with probability of misclassification less than 5% were used.

### RNA extraction and cDNA library preparation

To analyze the transcriptomes of protonemata, gametophores, and protoplasts and to validate the RNA-seq results, we extracted RNA as previously described [42]. The quality and quantity of the extracted total RNA was initially evaluated by electrophoresis in agarose gels with ethidium bromide staining. Quantification of the total RNA in the sample was carried out with the Quant-iT™ RNA Assay Kit (5–100 ng; Life Technologies, Carlsbad, CA, USA) in a Qubit fluorometer (Invitrogen, Carlsbad, CA, USA). The quality of the total RNA samples was evaluated using an Agilent RNA 6000 Nano kit and a 2100 Bioanalyzer (Agilent Technologies). The RNA was evaluated on the basis of peaks for 28S and 18S ribosomal RNA. The mRNA fraction was isolated using a MicroPoly(A)Purist™ Kit (Ambion, Carlsbad, CA, USA) according to the manufacturer’s recommendations. To achieve maximum removal of ribosomal and noncoding RNA from the sample, the procedure was repeated twice. The mRNA was quantified and the quality evaluated as described above. To generate a fragment library, about 500 ng mRNA of each sample was used. The mRNA fragment library was prepared with the SOLiD™ Total RNA-Seq Kit (Ambion) according to the manufacturer’s recommendations.

### SOLiD sequencing and sequence assembly

The sequencing of the mRNA fragment library was performed with a SOLiD 4 genetic analyzer (Applied Biosystems, Foster City, CA, USA) according to the manufacturer’s recommendations with both biological and technical repeats (gametophores: 2 biological and 2 technical repeats, protonema and protoplast: 3 biological and 2 technical repeats). We obtained 173, 197, and 204 million reads for the gametophore, protonema, and protoplast samples, respectively. The length of each read was 50 bp. The number of uniquely mapped filtered reads was 31, 36, and 38 million for gametophore, protonema, and protoplast samples, respectively. The reads were filtered with the SOLiD_preprocess_meanFilter_v2.pl utility using default parameters [reads with unread positions were rejected (hole filtering), as were those with average quality below 20].

As a reference, we used the *P. patens* genome v.1.6 (http://cosmoss.org) [43]. Reference genome mapping was performed with TopHat v2.0.7 software [44] using default parameters. To evaluate the gene expression level in RPKM, the produced .bam file was processed with the Cufflinks utility [45], and we used HTSeq to count the number of mapped reads for each gene. The number of uniquely mapped filtered reads was 31, 36, and 38 million for gametophore, protonema, and protoplast samples, respectively. We found evidence of expression of 18,412 coding sequences (CDS; at the level more than one read per million). To validate the accuracy and to evaluate the distortion that occurred during library preparation, the transcriptional levels of 17 genes were analyzed by quantitative real-time PCR (qRT-PCR). The Spearman correlation values of gene expression obtained by qRT-PCR and RNA-seq methods were 0.7, 0.7, and 0.8 for gametophore, protonema, and protoplast samples, respectively (see Additional file 1). For analysis of differential expression, the edgeR [46] package was used, and the analysis was performed according to the recommendations in the edgeR vignette. We used read count per gene data as input for edgeR. The genes up-regulated in protoplasts were identified using the following criteria for differential expression: a FDR level less than 0.05 and expression level difference between samples of at least four fold.

### Quantitative real-time PCR

Real-time PCR was performed using iQ SYBR Green Supermix (Bio-Rad) and the CFX96™ Real-Time PCR Detection System (Bio-Rad). Droplet digital PCR allows direct quantification of DNA molecules in a sample [47]. It was performed using ddPCR™ Supermix for Probes (Bio-Rad) and the QX100 system (droplet generator and droplet reader) along with a DNA Engine Tetrad 2 PCR machine (Bio-Rad). Real-time and ddPCR data were analyzed with CFX Manager and QuantaSoft (Bio-Rad) software, respectively. Primers and probes are listed in Additional file 2. PCR experiments were carried out using three biological and two technical replicates. We used the bootstrap method to determine the Pearson correlation coefficient.

### Analysis of photosynthetic activity of *P. patens* protonemata and protoplasts

To analyze changes in the photosynthetic activity of *P. patens* cells, we monitored the induction of chlorophyll fluorescence (Maxwell and Johnson, 2000; Adams and Demmig-Adams, 2004; Baker, 2008). The measurements were carried out with a FluorPen FP100 PAM-fluorometer (Photon Systems Instruments, Brno, Czech Republic). Fluorescence was measured in response to short (<50 μs) flashes of measuring light with average intensity not exceeding 0.1 μmol∙m^−2^∙s^−1^. Flashes of saturating light (3000 μmol∙m^−2^∙s^−1^) were 1 s long. The actinic light intensity ranged from 10 to 1000 μmol∙m^−2^∙s^−1^. The wavelength of measuring, saturating, and actinic light was 475 nm. Fluorescence was monitored in the range of 697–750 nm. To estimate the maximum quantum efficiency of photosystem (PSII) photochemistry, ${\varPhi}_{\max}^{\mathrm{PSII}}$, the sample was first adapted to darkness for 15 min. The operating quantum efficiency of PSII at light intensity X μmol∙m^−2^∙s^−1^, ${\varPhi}_{X\mu E}^{\mathrm{PSII}}$, depending on closing a part of PSII centers, was evaluated after cell adaptation to the given light intensity for 1 min after darkness or short-term treatment with light of lower intensity. The coefficient of non-photochemical quenching, *q*_*NPQ*_, was also evaluated while lighting cells after adaptation to darkness for 15 min.

## Results

### The *P. patens* gametophyte peptidome

**Figure 1:**
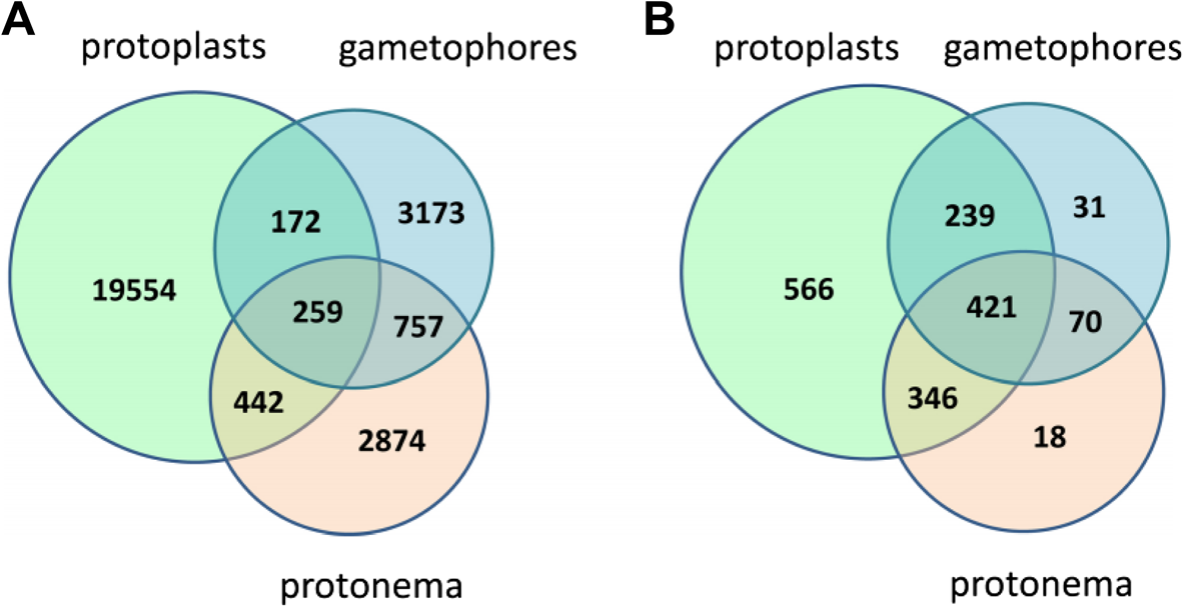
**Venn diagrams of peptide and precursor distributions in protonemata, gametophores, and protoplasts. А**, Peptide distributions. **B**, Precursor protein distributions.

Extracts of gametophores and protonemata were analyzed by tandem mass spectrometry (LC-MS/MS) against a uniref100 protein database for *P. patens*. Peptides ranging in size from 5 to 78 aa were identified (Additional file 3). A total of 4,361 peptide fragments of 761 precursor proteins were identified in gametophore extracts (Figure 1; Additional file 4). Since the majority of the identified precursors were annotated as predicted proteins, to assign their possible functions and localizations, we used BLAST homology analysis against the protein database of green plants (Viridiplantae). However, the most peptidogenic gametophore precursor proteins, A9U4I0, A9RXW5, and A9RXR3, with 144, 115, and 111 unique peptides respectively, were uncharacterized (see Additional file 4). Also, among the most peptidogenic precursor proteins were such major chloroplast proteins as A9SPD7 (photosystem I reaction center subunit IV, 52 native peptides) and A9U1R6 (outer envelope pore protein 16, 42 native peptides), as well as chaperone-like proteins, such as the A9SU24 (putative late embryogenesis abundant protein, group 3, 41 native peptides).

In protonema cells, mainly represented by chloronema cells, we identified 4,333 peptides that are fragments of 855 precursor proteins (see Additional file 5). Precursor proteins represented by a large number of peptides included major proteins like the large subunit of ribulose-1,5-bisphosphate carboxylase/oxygenase (RuBisCO), P34915 (giving rise to 111 native peptides), and elongation factor 1-alpha, A9RGA5 (80 native peptides), A9RX76 (chloroplastic fructose-bisphosphate aldolase, 48 native peptides), and A9SPD7 (photosystem I reaction center subunit IV, 47 native peptides), among others.

We analyzed the peptide abundance in peptidomes for several groups of precursor proteins. Gametophore and protonema peptidomes contain a prominent fraction of peptides (approximately 25% to the total peptidome) from the chloroplast precursor proteins (see Additional file 6). The share of fragments of mitochondrial (3% for protonema and 10% for gametophores) and nuclear (4% for protonema and 5% for gametophores) precursor proteins, as well as of those proteins involved in translation (ribosome proteins, elongation factor alpha, etc.; 8% for protonema and 3% for gametophores), was also rather high. Since LEA proteins were among the most peptidogenic precursor proteins in gametophores, we evaluated their contribution to the peptidome as well. LEA protein content was higher in gametophores than in protonema (164 native peptides in gametophores vs 38 peptides in protonema), while peptides of other chaperon-like proteins, such as the heat shock proteins, were more abundant in protonema (125 peptides in protonema vs 66, in gametophores) (see Additional file 6).

### Dramatic changes in the *P. patens* protoplast peptidome

*Physcomitrella patens* protoplasts are released after treating young protonema tissue with Driselase, a natural enzyme mixture containing laminarinase, xylanase, and cellulase activities [48]. This process severely stresses plant cells through the loss of the cell wall, as well as through the effects of the compounds in the crude Driselase preparation. We also considered that, as protoplasts are a good model for studying reprogramming of somatic plant cells [35], peptidomic information must be essential to describe the metabolic landscape.

We found that *P. patens* protoplast peptidome comprised 20,427 peptides, ranging in size from 6 to 78 aa (Additional file 3), that were fragments of 1,572 precursor proteins (see Additional file 7). Notably, a considerable part of the peptides in protoplasts differed from each other by deletion of either the C- or N-terminal amino acid. Apparently, the “peptide ladders” result from degradation of the peptides by amino- and carboxypeptidases (Additional file 3).

In the protoplast peptidome, we identified a large number of peptides derived from chloroplast proteins (see Additional file 6). The large subunit of RuBisCO underwent the most severe degradation. Among the other highly represented precursor proteins were photosystem I reaction center subunit II-2, RuBisCO activase, carbonic anhydrase, elongation translation factor 1-alpha, lipoxygenase, and plastocyanin. All these data point to intensive protein degradation in protoplasts. There is evidence that chloroplast proteins like RuBisCO and, particularly, RuBisCO activase are the main targets for cysteine protease in vacuoles of plant cells [49]. We tested whether the observed peptides were generated in chloroplasts. In chloroplasts isolated from protoplasts, we identified 82 unique peptides that were fragments of 21 precursor proteins (see Additional file 8); only three of the peptides were fragments of the large subunit of RuBisCO, one of the most abundant proteins in cell. These data confirm that the major chloroplast proteins are degraded outside the intact chloroplasts.

To examine the factors involved in this marked difference in the amount/diversity of peptides, we tested the effects of Driselase at a concentration lower than that used to isolate the protoplasts. When protonema tissue was treated with 0.025% (w/v) Driselase solution, protoplasts did not form. However, treatment of protonemata with Driselase at this concentration resulted in 2.5 times more native peptides (Additional file 9). This finding is indicative of the “biotic” stress that protonema cells undergo when being treated with Driselase.

To evaluate the fraction of peptides that could be isolated from dead cells, we assessed the viability of protoplasts using the Trypan blue dye. In the analysis, we were not able to identify a substantial number of stained cells (data not shown). Such results indicate that dead or dying protoplasts did not significantly affect of our results. Neither did we detect a significant number of peptides in the protoplast wash solution (data not shown).

Earlier, functionally active proteins were shown to potentially have encrypted sequences possessing antimicrobial activity [16]. To evaluate the antimicrobial potential of the identified peptides, we used the Antimicrobial Sequence Scanning System (AMPA) [41]. We identified 117 endogenous peptides that might have antimicrobial activity, 81 of which were unique to protoplasts, 11 to protonemata, and 27 to gametophores (see Additional file 10).

### Functional analysis of precursor proteins

**Figure 2:**
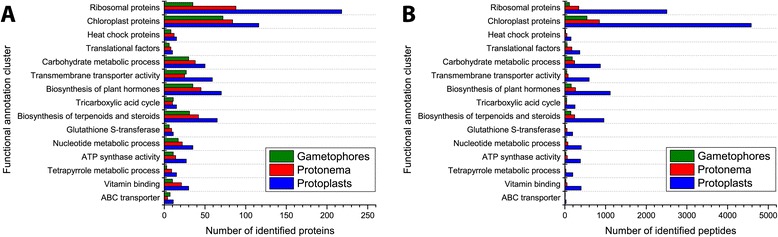
**Distribution of the clusters of precursor proteins. A**, Distribution of the clusters by the number of peptides identified in the gametophores, protonemata, and protoplasts. **В**, Distribution of the clusters by the number of identified precursor proteins in the gametophores, protonemata, and protoplasts. Statistically significant (p < 0.05) clusters of precursor proteins were grouped by the DAVID web service based on the default functional annotation database set.

We used the identified precursor proteins of peptides to carry out functional analysis using the Database for Annotation, Visualization and Integrated Discovery (DAVID) (http://david.abcc.ncifcrf.gov/). After 1,691 of the precursor proteins were correlated with proteins in the DAVID database, we performed clustering with a high threshold to decrease the number of clusters. We obtained 15 clusters comprising 1,030 proteins (Additional file 11). Precursor proteins localized in chloroplasts and ribosome structural components were the two most represented clusters in all three life forms (Figure 2). Many other precursor proteins were involved in the processes of carbohydrate metabolism, transmembrane transporter activity, and biosynthesis of plant hormones, terpenoids, and steroids. The number of precursor proteins referred to the ribosomal protein cluster was highest in protoplasts. However, in the protoplast peptidome, more peptides were derived from chloroplast precursor proteins than from ribosomal precursor proteins. The total amount of endogenous peptides identified in protoplasts differed considerably compared to gametophores or protonemata, indicating that peptide generation depends on cell type. For example, in protoplasts, we identified 4,580 peptides derived from chloroplast precursor proteins, more than five times the number in gametophores (543 peptides) or protonemata (848). The number of identified precursor proteins in protoplasts related to chloroplasts (116 proteins) was only about 1.5 times greater than those in gametophores (72) or protonemata (84).

### The change of gene expression profiles in *P. patens* protoplasts

To evaluate the transcription levels of the identified peptide precursor proteins and to examine what cellular mechanisms were activated to cause the significant differences observed in the protoplast peptidome, we performed transcriptome analysis of gametophores, protonemata, and protoplasts. We identified 1936 genes up-regulated in protoplasts (Additional file 12). Analysis of Gene Ontology (GO) terms showed that, in protoplasts, the transcriptional levels increased of genes involved in responses to different stress factors, e.g., abiotic stimulus (GO:0009628), cold (GO:0009409), temperature (GO:0009266), and oxidative stress (GO:0006979; Additional file 13; Additional file 14). We identified several genes that participate in jasmonic acid (JA) biosynthesis. In addition, the transcription levels of eight WRKY transcriptional factors involved in gene regulation in a range of processes, including biotic and abiotic stresses, senescence, and different developmental processes, increased [50].

We also examined the differentially expressed genes that could contribute to the protoplast peptide pool. There was a significant increase in the transcription of Pp1s166_98V6, encoding putative proteasome activating protein 200 (PA200). Also, a number of genes encoding proteases, for example Pp1s78_186V6 (*subtilisin-like serine protease 2*), Pp1s112_240V6 (*subtilisin-like serine protease 3*), Pp1s39_149V6 (a homolog of mitochondrial protease *FtsH3*), and Pp1s5_15V6 (a homolog of protease *Lon1*), showed higher transcription levels in protoplasts. In addition, we observed increased transcription of genes responsible for ubiquitin-mediated protein breakdown, e.g. RING/U-box superfamily protein and E3 ubiquitin ligase family protein (see Additional file 15 online). Interestingly, we identified significant increases in the transcription levels of the peptide transporter genes, such as Pp1s114_7V6 (*peptide transporter 1*, log_2_ = 6.3) and Pp1s72_96V6 (*peptide transporter 5*, log_2_ = 2.3). We also noted an increase in the transcription of Pp1s1_60V6 (*AtTAP2*), a plant analogue of *TAP* (*Transporter associated with Antigenic Processing*), which transports peptides generated by the proteasome complex into the endoplasmic reticulum where they are loaded onto a newly synthesized MHC class I complex in humans [51]. This fact could be related to an increase in the pool of free amino acids and/or oligopeptides or to enhanced direct transport of peptides from the cell.

### Comparative quantitative analysis of precursor proteins

We tried to determine whether there was any correlation between the quantity of precursor proteins in a cell and the amount of endogenous peptides in its peptidome. We roughly estimated the quantitative relation of precursor proteins in protonemata vs gametophores and protonemata vs protoplasts. We correlated the protein abundance with the transcription level and the number of endogenous peptides of corresponding precursor proteins. In the protonemata vs gametophores case, we can see a week correlation between precursor protein abundance and their transcriptional levels as well as with the number of endogenous peptides (Additional file 16). Yet, when comparing protonemata and protoplasts, we found no correlation between transcription levels and protein abundances nor between the number of endogenous peptides and the abundances of the corresponding proteins (Additional file 17).

The detailed analyses of certain precursor proteins showed that even when cells of two developmental states had equal abundances of a protein, the number of endogenous peptides could differ significantly (Additional files 18 and 19). As an example, a range of stromal chloroplast proteins, such as P34915 (ribulose bisphosphate carboxylase large chain), A9TC11 (phosphoglycerate kinase, chloroplastic), A9T3W5 (small chain of ribulose bisphosphate carboxylase, chloroplastic), and A9TBP0 (ribulose bisphosphate carboxylase/oxygenase activase 1, chloroplastic), showed very different peptide contents in the two developmental stages (Additional files 18 and 19). Precursor proteins such as A9U1H4 (probable linoleate 9S-lipoxygenase 4), A9RD61 (major allergen Mald 1), and A9U188 (glycerate dehydrogenase) produced more endogenous peptides in protonemata than in gametophores although they were less abundant in the former. For such precursor proteins as A9SCV0 (glutathione S-transferase F9), A9RT52 (5-methyltetrahydropteroyltriglutamate-homocysteine methyltransferase), A9RWS2 (5-methyltetrahydropteroyltriglutamate-homocysteine methyltransferase), A9TIY2 (glutamine synthetase, chloroplastic), and a range of others, we observed not only increased levels of proteins and peptides, but also higher transcription of the corresponding genes in protonemata. Only a few precursor proteins, such as A9RPL4 (ribosomal protein S25 family protein) or A9RWN4 (small nuclear ribonucleoprotein family protein) showed a decrease of protein abundance in protoplasts with an increase of the number of endogenous peptides from corresponding proteins in the peptidome, which may indicate degradation of these proteins (Additional file 18). In other cases, we observed an increase of endogenous peptide number along with an increase in protein abundance. Examples include the precursor proteins A9RAS2 (chlorophyll a-b binding protein, chloroplastic), A9SL09 (photosystem I reaction center subunit VI, chloroplastic), A9SX31 (superoxide dismutase [Cu-Zn], chloroplastic), A9T3W5 (ribulose bisphosphate carboxylase small chain, chloroplastic), and others (Additional file 18). Also, we did not observe any significant changes in the transcriptional levels of corresponding genes in protonemata and protoplasts.

**Figure 3:**
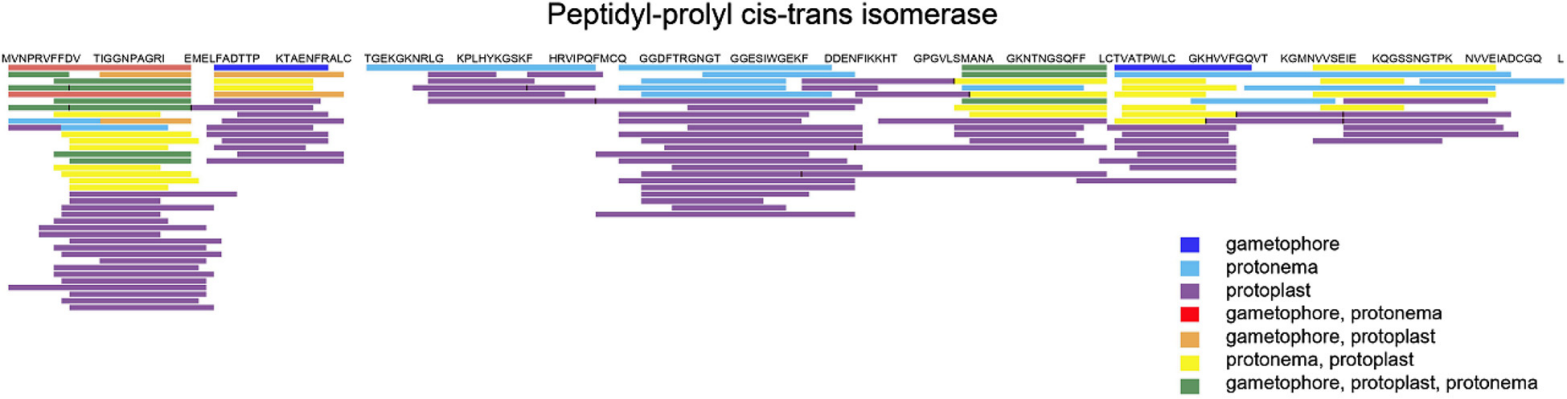
**Peptide alignment of a precursor protein A9TK88 (peptidyl-prolyl** ***cis*** **-trans isomerase).**

Thus, our results showed that the degradation pathway of a protein can be different in gametophores, protonemata, and protoplasts. The peptide alignments of several most represented proteins supported this assumption (for example, see Figure 3).

### Photosynthetic activity changes during protoplast isolation

As stated earlier, most precursor proteins identified in protoplasts were chloroplast proteins. Therefore, we analyzed the changes in protoplast photosynthetic activity during their isolation from protonema cells.

In cells exposed to light, the operating quantum efficiency of PSII photochemical activity $\left({\varPhi}_{\mathrm{light}}^{\mathrm{PSII}}\right)$ in protonema cells started to diminish at the beginning of maceration and, by the time protoplasts were released from protonema tissue, both the maximal Φ^PSII^ value ${\varPhi}_{\max}^{\mathrm{PSII}}$ and, especially, the operating value at moderate light intensity (100 μmol∙m^−2^∙s^−1^), ${\varPhi}_{100\mu E}^{\mathrm{PSII}}$ (Additional file 20) were significantly lower than in intact protonema cells. The decrease in ${\varPhi}_{\mathrm{light}}^{\mathrm{PSII}}$ indicates disrupted electron outflow from PSII, which can be due to suppressed uptake of photosynthetic light-phase outputs (ATP and NADPH). Under normal conditions, the main consumer of ATP and NADPH in photosynthetic cells is the carbon dioxide fixation system; thus, damage to Calvin cycle enzymes can reduce ${\varPhi}_{\mathrm{light}}^{\mathrm{PSII}}$ (both ${\varPhi}_{\max}^{\mathrm{PSII}}$ and, especially, ${\varPhi}_{100\mu E}^{\mathrm{PSII}}$). This result is in agreement with the observed degradation of the large subunit of RuBisCO. Notably, Φ^PSII^ of protonemata also diminishes upon treatment with low Driselase concentrations (which do not lead to protoplast isolation, but nevertheless degrade some cell proteins). Thus, the decrease in Φ^PSII^ was not related to mechanical injury of cells or chloroplasts.

## Discussion

Little is known about the qualitative and quantitative compositions of the peptide pools that result from protein degradation in cells. The processes of protein synthesis and degradation are constantly ongoing in cells. The rate of protein turnover, as well as the composition of proteins subjected to degradation, changes in response to many factors [52,53]. We found that the two developmental stages of *P. patens*, namely protonemata and gametophores, as well as protoplasts, contain thousands of endogenous peptides resulting from degradation of functionally active proteins. We observed differences in the peptide pools of the three moss cell types, which could reflect both their different protein contents and differences in the regulatory mechanisms of degradation. Our attempt to elucidate the correlation between protein abundance and the amount of endogenous peptides revealed that degradation pathways of a given protein could be different for gametophores, protonemata, and protoplasts. This result may indicate that the identified endogenous peptide products of protein degradation are not a mere “noise” but may play some role in the cell. Thus, this area offers a vast field for further research.

### Peptide pools in moss gametophores and protonemata differ

In the peptidome of young and growing protonema tissue, we identified a large number of peptides that are derived from chloroplast proteins. The most abundant precursor proteins in protonemata were major cell proteins such as elongation factor 1a and the large subunit of RuBisCO, as well as fructose-bisphosphate aldolase. The predominance of peptides derived from chloroplast proteins might be due to the rapid growth of protonema cells, resulting in intensive metabolism by proteins related to cellular energy processes. For example, Nelson et al. demonstrated that some major proteins involved in photosynthesis turn over at an above-average rate. Also, chloroplast proteins can serve as a source of amino acids, and in growing tissues RuBisCO acts as a nitrogen source [52]. We found that proteins involved in carbohydrate metabolism, transmembrane transporter activity, and biosynthesis of plant hormones, terpenoids, and steroids are also among the most peptidogenic precursor proteins. We assume this fact correlates with the rate of turnover of these proteins in cells. Besides, as shown by Nelson et al., proteins involved in tetrapyrrole metabolism also degrade rapidly. We identified a separate cluster of such precursor proteins that generated the highest numbers of peptides in protoplasts (see Figure 2).

In addition to chloroplast protein fragments, the peptidome of the mature gametophyte stage, the gametophore, also contains large amounts of peptides from chaperone- and stress-related proteins, such as LEA proteins, aquaporins, AWPM-19-like, and formate dehydrogenases (Additional file 6). A possible explanation could be the fact that gametophores, unlike protonemata, grow in the air environment, and water balance regulation is therefore important in this tissue. Notably, Widiez et al. detected activation of genes responsible for the response to lack of water as early as at the stage of gametophore development from protonemata and hypothesized that such changes play protective roles [54]. Our data on the elevated transcription levels of these genes, as well as the greater numbers of endogenous peptides derived from these proteins, support that hypothesis. One may suppose that an increase in a protein’s representation in the proteome leads to more endogenous peptides—products of hydrolysis of that protein—in the peptidome. However, until we know the rate of the protein’s turn over, we cannot reliably conclude whether the elevated peptide levels are due to rapid turnover of the protein or to its targeted degradation.

LEA proteins were first discovered in plant seeds [55,56]. Plants biosynthesize LEA proteins in response to drought or abscisic acid [57]. These proteins protect other cellular proteins from denaturation and aggregation under low water content [58]. In *P. patens*, LEA proteins are expressed at a basal level in gametophores, which could be related to protection from water stress [30]. We do not know whether endogenous peptides of chaperone-like proteins like LEA play protective roles in the moss cells, although previous research shows that short LEA protein fragments can independently serve as chaperones [59]. Peptides of the LEA protein family were also identified in both protonemata and protoplasts, but the number of unique peptides for these proteins was higher in gametophores. According to Lienard et al. [60], the *P. patens* aquaporin genes *PIP2-1* and *PIP2-2* are expressed only in gametophore cells and not in protonemata, consistent with our data that native peptides derived from *PIP2-1* and *PIP1-4* are present only in gametophores. Such agreement between mass spectrometry and transcriptomic data holds for many precursor proteins, peptides of which can be identified only in gametophores or protonemata. Quantitative analysis of peptides in protonema and gametophore cells will be the subject of our further research.

### Peptidome of *P. patens* protoplasts

**Figure 4:**
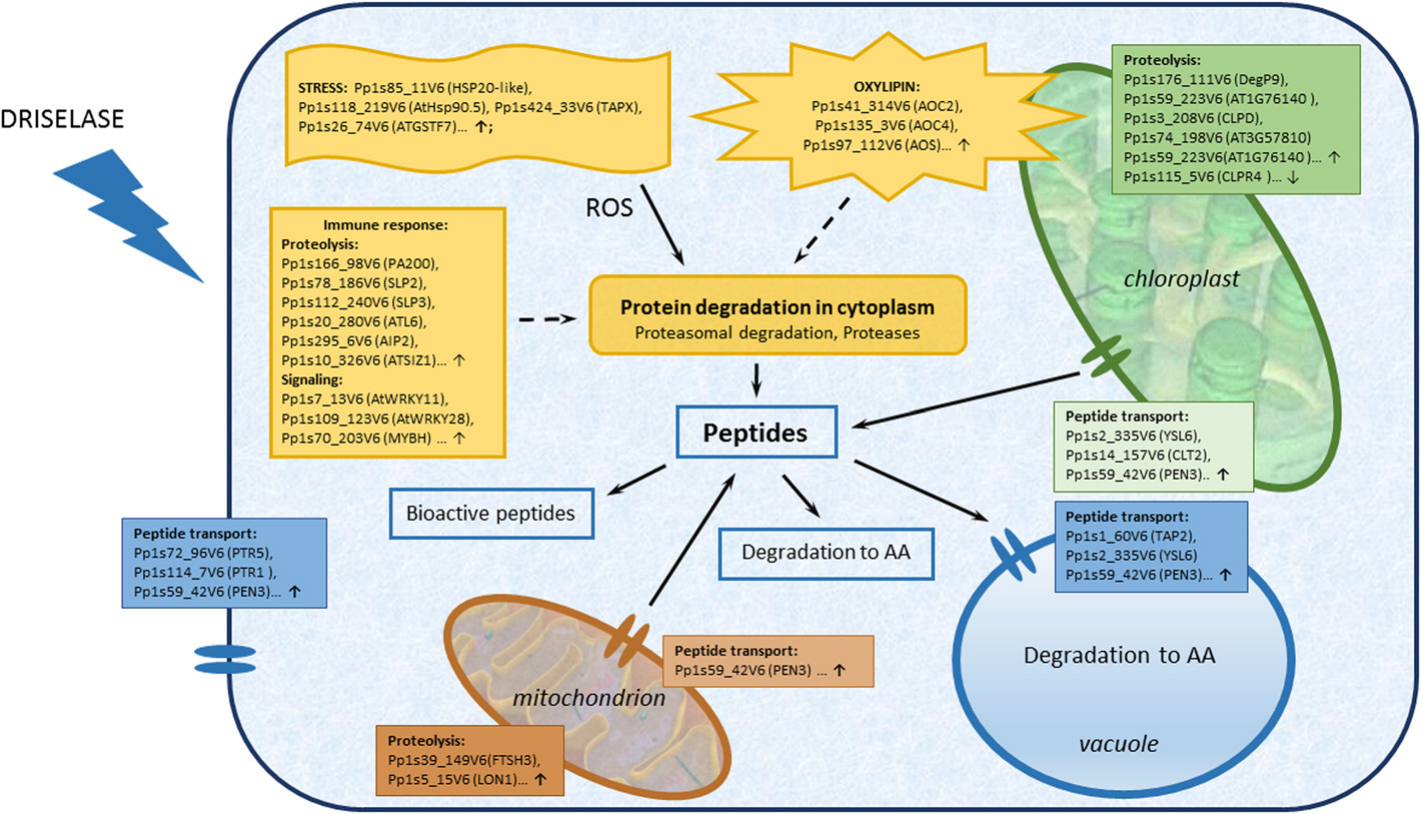
**Peptide generation process in protoplasts.** We used transcription profiling data to construct a model describing the peptidogenesis in protoplasts. Up-regulated genes associated with factors affecting the protein degradation, such as stress response leading to increase in ROS, immune reactions, and synthesis of stress hormones, are indicated. We inferred that some peptides are degraded to amino acids, while another fraction can be biologically active. The scheme also indicates up-regulated genes encoding proteases and peptide transporters, which also can contribute to the cell peptidome. Arrows show some genes that were up-regulated 2-fold. A complete list of genes and quantitative data for this experiment are given in Additional file 12.

We suggest several hypotheses to explain the dramatic increase in the numbers of precursor proteins, as well as of protoplast-specific peptides, that occur in the protoplast peptidome: 1) an immune response leading to specific degradation of cell proteins; 2) an increase in protein degradation rate induced by stress; and 3) protein degradation due to programmed cell death (Figure 4). We analyzed the differentially expressed genes of protonemata and protoplasts to elucidate what molecular mechanisms could be responsible for the significant changes observed in the protoplast peptidome. In addition to the reactions to abiotic stress, which may be associated with the change in cellular form, we also observed increased expression of genes that respond to biotic stress. This result points to the complex nature of stress experienced by cells during protoplast isolation. Previously, Xiao et al. analyzed the protoplast transcriptome during regeneration [35]. According to their findings, the expression of some stress genes as well as of genes responsible for biosynthesis of a range of phytohormones, increases in protoplasts, while photosynthetic pathways are inhibited. However, comparative analysis of protonema and protoplast transcriptomes was not performed in that work.

Cell wall degradation, active elicitors, and the contents of the disrupted cell could induce immune reactions during protoplast isolation [61]. According to our findings, protoplasts have significantly increased expressions of allene oxide cyclase 2 (*АОС2*, Pp1s41_314V6), allene oxide cyclase 4 (*АОС4*, Pp1s135_3V6), and allene oxide synthase (*AOS,* Pp1s97_112V6) (see Additional file 12), which participate in JA biosynthesis [62,63]. *Physcomitrella patens* protonemata and gametophores do not contain JA, but instead use its precursor 12-oxo-phytodienoic acid [63]. However, *P. patens* responds to methyl jasmonate by reducing moss colony growth and rhizoid length, suggesting that jasmonate perception is present in mosses [64]. Because the synthesis of oxylipins in plants is related to biotic stress, findings like these constitute additional evidence favoring the inference that peptidome rearrangement in protoplasts can be related to a severe “biotic” stress due to the effects of Driselase on cells. JA causes degradation of specific proteins and synthesis of specific peptides and promotes senescence [65,66]. Plant defense response is related to the decreased expression genes involved in photosynthesis and is also accompanied by a slowdown in plant growth [67]. However, the physiological importance and mechanisms of the phenomenon have been poorly studied thus far. Our data on photosynthetic activity of moss cells during protoplast isolation show that the decrease in the photosynthesis activity of protonemata treated with Driselase depends on the concentration of the enzyme applied to protonemata and correlates with the increase in the amount of peptides—the products of chloroplast protein degradation. In other words, the observed significant increase in the number of native peptides from chloroplast proteins in protoplasts might be linked to important physiological effects.

Growth of the peptide number in plants that underwent biotic stress can also be due to the protective or signaling role of such peptides [4,17]. Whether the observed effect of the dramatic peptidome change in protoplasts is the result of non-specific degradation or whether it serves a protective function is not yet clear. Peptides, such as Systemin, HypSys, GmSubPep, GmPep914, AtPeps, and inceptins induce plant defense mechanisms under biotic stress [20,68]. Three of these, GmSubPep, GmPep914, and inceptin, are produced during the breakdown of cellular proteins that have their own metabolic functions unrelated to stress reactions. Furthermore, biotic stress leads to proteolysis of specific proteins, yielding endogenous peptide elicitors [66]. We evaluated the antimicrobial potential of the identified peptides and revealed 117 endogenous peptides with high potential antimicrobial activity. Of these, 81 were identified in protoplasts. Induction of antimicrobial peptides under the stress conditions used here sheds light on the probable function of peptide pools in *P. patens*. However, checking the antimicrobial activity of such peptides demands further research. This hypothesis agrees with the increase in transcription levels of peptide transporters, which can play an important role in bidirectional transport of peptides. Interestingly, a range of peptide transporters, such as PEN3 (Pp1s59_42V6) and CLT2 (Pp1s14_157V6), can function in transportation of such peptides and are induced under biotic stress.

Another mechanism influencing protein degradation is the effect of stress factors. Stress conditions affect degradation of cell proteins and, consequently, can significantly change the cell peptidome. Severe stress in protoplasts could be detected by the increased expression of genes participating in protection from reactive oxygen species (ROS), such as Pp1s424_33V6, *thylakoidal ascorbate peroxidase*; Pp1s26_74V6, *glutathione S-transferase PHI 9*, and others. An increase in ROS level can lead to oxidation and subsequent protein degradation. Degradation of RuBisCO might be triggered by oxidation of critical cysteine residues in the protein [69]. Our data indicate that, in protoplasts, peptide generation from stromal and membrane thylakoid proteins occurs outside of chloroplasts. In protoplasts, none of the genes encoding chloroplast proteases exhibited elevated transcription levels, except for the gene encoding Pp1s3_208V6 (ATP-dependent Clp protease regulatory subunit), which regulates the activity of the Clp protease complex in chloroplasts (Additional file 15). At the same time, the level of transcription of the Pp1s115_5V6 gene encoding the R4 subunit of Clp protease complex decreased. The existence of membrane compartments containing RuBisCO (called RuBisCO vesicular bodies, RVB) in the cytoplasm also suggests that there is a relationship between protein degradation systems in the chloroplasts, cytosol, and vacuoles [49] [70]. Today, little is known about the mechanisms of degradation of other chloroplast proteins, especially as part of the peptide generation process. The available data on stress-related degradation of chloroplast membranes with subsequent release of proteins to the cytoplasm suggests the hypothesis that they may be degraded outside chloroplasts under stress conditions [71].

For some chloroplast proteins, such as A9S1E1 (photosystem I reaction center subunit XI), A9S6S7 (LHCB2, light harvesting complex of photosystem II), and A9TIY2 (GLN2, glutamine synthetase) we observed a decrease in their abundance and an increase in the number of their endogenous peptides. However, for the majority of chloroplast proteins, we observed a positive correlation between protein and endogenous peptide abundances. This finding can be explained by an elevated rate of protein turnover, leading to enhanced degradation and, consequently, more peptides from these proteins.

Although little is known about the generation of peptides *en masse* in plant cells, biotic stress triggers a number of cellular destruction mechanisms. For example, the hypersensitive reaction involves programmed cell death and can lead to massive protein degradation [72]. Along with that, a number of proteases are induced by biotic stress [73], which also could lead to peptide generation and a significant change in the peptide pool of protoplasts. Also, we detected an increase in transcription levels of the genes of heat shock proteins (HSPs), such as Pp1s97_106V6 (HSP 21) and Pp1s3_114V6 (HSP17.6II), which can indicate the initialization of cell death mechanisms [74], similarly to apoptosis in animal cells [75,76].

### Mechanisms of peptide generation in plant cells

The identified peptide pools apparently result from protein degradation processes in the cell. What is the nature of the observed proteolytic degradome of plant cells? A number of studies have shown that plant cells, like animal cells, have a complex protein degradation system. Plants have two main ways to degrade intracellular proteins: the proteasome-mediated pathway and autophagy [77].

The proteasome is a proteolytic complex that degrades short- and long-lived intracellular proteins. Plants have two types of proteasomes, 20S and 26S, that are responsible for ubiquitin-dependent and ubiquitin-independent proteolysis, respectively [78]. In proteasomes, proteins are degraded to peptides of 2–25 residues. The generally accepted view is that peptides generated by proteasomes are labile in the cytoplasm and hydrolyzed rapidly to amino acids [79]. However, there is some evidence that, in plants, these peptides can be transferred to the vacuole for further degradation [51]. In mammals, a set of proteases degrades proteasome-generated peptides [79,80]. In plants, a similar set of proteases with the same function was also discovered [81-83]. In a previous report, oligopeptides generated by proteasomes were not found in cell extracts [84] possibly because of rapid degradation *in vivo* [80]. However, the tools available at that time did not allow accurate identification of peptide pools. Still, accumulation of peptide fragments in a cell was assumed to be undesirable, because it may prevent protein-to-protein interactions and the peptides could possibly aggregate and become toxic [85,86]. However, we identified several thousand native peptides in gametophore and protonema cells and about 20,000 peptides in protoplasts. The results of our study provide ground for a more detailed description of peptide generation in *P. patens*. Examining peptide families derived from individual proteins allows several common principles of protein degradation to be outlined. The original amino acid sequence of the protein is clearly divided into segments 15–25 amino acid long that give rise to sets of structurally related peptides. We believe that primary proteolysis occurs at sites located between these segments, presumably within proteasomes. After the primary segments are released from the proteasome, they are further degraded to smaller peptides by amino- and carboxypeptidases [81-83]. Degradation products of peptidyl-prolyl cis-trans isomerase shown in Figure 3 serve as an example.

Proteins or peptides also can be degraded by a large number of other unspecified proteases. Plant genomes encode hundreds of proteases that belong to a dozen unrelated families [87]. The biological roles of the majority of proteases are unknown, but the very mechanism of their effects on their substrates suggests that they may also contribute to peptide pool formation in the cell [87].

Stress conditions might modify the above-described picture. Stimuli such as infection, drought, cold, and ultraviolet light result in increased ROS in the cell [52]. The elevated ROS levels result in oxidized protein, which might be cytotoxic [88]. In animal cells, proteasomes recognize and degrade oxidized proteins in the cytosol, nucleus, and endoplasmic reticulum, thus protecting the cell from their cytotoxicity [89]. *In vitro*, the 20S proteasome recognizes and degrades the oxidized proteins while components of the ubiquitin-dependent 26S proteasome pathway become inactive [90]. In plants, severe stress results in excess amounts of proteolytic substrate, and oxidation stress directly inhibits the 26S proteasome degradation pathway [78,91].

We assume that the mechanisms described above can be responsible for the formation of peptide pools in cells. We suppose, under stress conditions, proteins undergo active proteolysis, leading to rapid and extensive changes in the peptidome. We found that expression of some genes encoding proteases and proteins involved in ubiquitin-dependent degradation of proteins increased in protoplasts. For example, transcription of the Pp1s166_98V6 gene encoding PA200 increased in protoplasts. PA200 is a non-ATPase activator of 20S particles of proteasomes, thus it could be responsible for the increase in hydrolysis of small peptides [92]. Notably, the level of transcription of eleven ubiquitin ligase E3 genes belonging to RING/U-box class and involved in proteasome-dependent protein degradation was also higher in protoplasts (Additional file 15). These proteins are known to participate in responses to abiotic and biotic stresses in plants [93,94]. Interestingly, expression of PA200, as well as subtilisin-like serine protease 2 (Pp1s78_186V6) and subtilisin-like serine protease 3 (Pp1s112_240V6), is induced by JA in *Arabidopsis* [95,96]. This fact agrees with our data on increased transcription of genes involved in oxylipin synthesis. Besides, the transcription levels of mitochondrial proteases, such as Pp1s39_149V6 (a homolog of mitochondrial protease FtsH 3) and Pp1s5_15V6 (a homolog of mitochondrial protease Lon1), is increased in protoplasts, perhaps indicating active proteolysis in mitochondria. At the same time, transcription of a number of genes encoding proteases, such as Pp1s90_35V6 and Pp1s24_95V6 (aspartyl protease family proteins), Pp1s8_260V6 and Pp1s283_60V6 (prolyl oligopeptidase family proteins), Pp1s233_64V6 (Peptidase C78), Pp1s58_185V6 (serine carboxypeptidase-like 50), Pp1s113_206V6 (carboxypeptidase D), is lower in protoplasts. Some proteases with higher expression in protoplasts localize in chloroplasts, for example, Pp1s8_260V6 and Pp1s283_60V6 (prolyl oligopeptidase family proteins) and Pp1s233_64V6 (Peptidase C78). This finding probably indicates the fine regulation of the processes of protein degradation and peptidome generation in plant cells.

## Conclusions

In conclusion, our results show that moss cells contain extended peptide pools that are hydrolysis products of cell proteins. The peptide pool composition depends on the type of tissue yet always contains peptides derived from the major chloroplast proteins. We observe no correlation between protein abundance, its transcription level, and the amount of endogenous peptides. Active peptidogenesis in protoplasts is probably due to a range of mechanisms, with stress during isolation and immune reaction to Driselase treatment being the key ones. Eighty-nine peptides of protoplasts possess high antimicrobial potential. Genes involved in JA synthesis, as well as those associated with biotic stress, had increased transcription levels in protoplasts. Changes in the peptidome in protoplasts are accompanied by suppression of photosynthetic activity. In our future research, we aim to study which mechanisms of degradation are responsible for the formation of endogenous peptide pools in cells, evaluate biological activity of the peptides, and study the effects of hormones on peptidome formation.

### Availability of supporting data

The mass spectrometry proteomics data have been deposited in the ProteomeXchange Consortium (http://proteomecentral.proteomexchange.org) via the PRIDE partner repository [97] with the dataset identifier PXD000227 and doi:10.6019/PXD000227. Sequence data described in this article can be found in the ArrayExpress (http://www.ebi.ac.uk/arrayexpress/) database under accession number E-MTAB-1637.

## Additional files

Additional file 1:
**Correlation between RNA-seq data and quantitative real-time PCR analysis.**
Additional file 2:
**List of primers and probes used in quantitative real-time PCR.**
Additional file 3:
**List of peptides identified in gametophores, protonemata, and protoplasts.**
Additional file 4:
**List of precursor proteins of endogenous peptides identified in gametophores.**
Additional file 5:
**List of precursor proteins of endogenous peptides identified in protonemata.**
Additional file 6:
**Ratios of endogenous peptides derived from eight groups of precursor proteins in peptidomes of gametophores, protonemata, and protoplasts.**
Additional file 7:
**List of precursor proteins of endogenous peptides identified in protoplasts.**
Additional file 8:
**List of precursor proteins of endogenous peptides identified in intact chloroplasts.**
Additional file 9:
**Venn diagram showing the amount of peptides identified in protonemata treated with 0.025% (w/v) Driselase compared with untreated protonemata (control).**
Additional file 10:
**List of endogenous peptides with high antimicrobial potential (AMPA results).**
Additional file 11:
**Functional annotation of precursor proteins using the DAVID service.**
Additional file 12:
**Differentially expressed (DE) genes in protoplasts compared to protonemata.**
Additional file 13:
**GO terms of up-regulated genes in protoplasts compared to protonemata.**
Additional file 14:
**Distribution of differentially expressed (DE) genes over the clusters obtained with the DAVID tools.**
Additional file 15:
**Differentially expressed (DE) genes related to protein degradation in protoplasts compared to protonemata.**
Additional file 16:
**Change in protein abundance across gametophores and protonemata.**
Additional file 17:
**Change in protein abundance across protoplasts and protonemata.**
Additional file 18:
**Quantitative relationship of precursor proteins in protonemata versus gametophores.**
Additional file 19:
**Quantitative relationship of precursor proteins in protonemata versus protoplasts.**
Additional file 20:
**Time-courses of maximal** $\left({\varPhi}_{\max}^{\mathrm{PSII}}\right)$ **and operating value at moderate light intensity (100 μmol∙m** ^**−2**^ **∙s** ^**−1**^ **;** ${\varPhi}_{100\mu E}^{\mathrm{PSII}}$) **of quantum efficiency of photosystem II (PSII) photochemical activity in** ***Physcomitrella patens*** **protonema cells under treatment with 0.5%, 0.025%, and 0.0025% Driselase solution.**

## Abbreviations

qRT-PCR: Quantitative real-time PCR
RuBisCO: Ribulose-bisphosphate carboxylase
LEA: Late embryogenesis abundant protein
CDS: Coding sequences
ROS: Reactive oxygen species
NADPH: Nicotinamide adenine dinucleotide phosphate
ATP: Adenosine-triphosphate
JA: Jasmonic acid
PSII: Photosystem II
