# Supplementary material for: Specific pools of endogenous peptides are present in gametophore, protonema, and protoplast cells of the moss Physcomitrella patens
Source: BMC Plant Biol. 2015 Mar 15;15:87. doi: 10.1186/s12870-015-0468-7 (PMC4365561; doi:10.1186/s12870-015-0468-7)
Supplement: Additional file 1: — Correlation between RNA-seq data and quantitative real-time PCR analysis. [file 12870_2015_468_MOESM1_ESM.pdf]

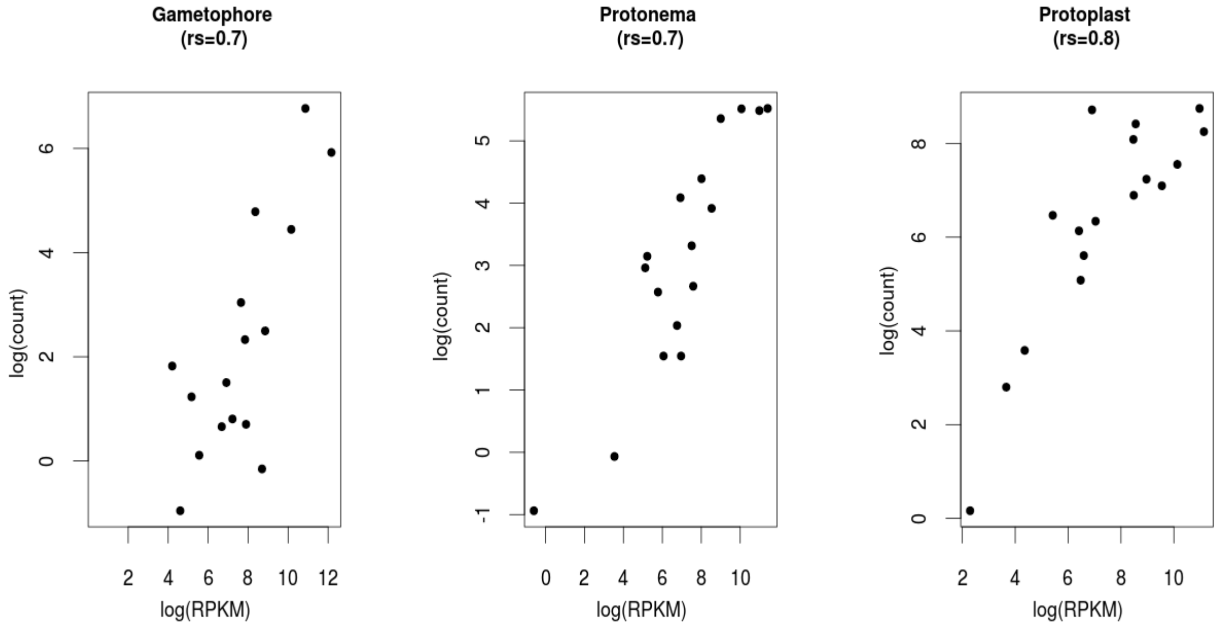

Additional file 1. Correlation between RNA-seq data and quantitative real-time PCR analysis. To validate the accuracy and to evaluate the distortion that occurred during library preparation, the transcriptional levels of seventeen genes, which had only one transcriptional variant and no paralogs in their genome, were analyzed by quantitative real-time PCR (qRT-PCR). The Spearman correlation values of gene expression obtained by qRT-PCR and RNA-seq methods were 0.7, 0.7 and 0.8 for gametophore, protonema and protoplast samples, respectively. We used the median value of different repeats of RNA-seq for each sample.
