# Supplementary material for: Specific pools of endogenous peptides are present in gametophore, protonema, and protoplast cells of the moss Physcomitrella patens
Source: BMC Plant Biol. 2015 Mar 15;15:87. doi: 10.1186/s12870-015-0468-7 (PMC4365561; doi:10.1186/s12870-015-0468-7)
Supplement: Additional file 6: — Ratios of endogenous peptides derived from eight groups of precursor proteins in peptidomes of gametophores, protonemata, and protoplasts. [file 12870_2015_468_MOESM6_ESM.pdf]

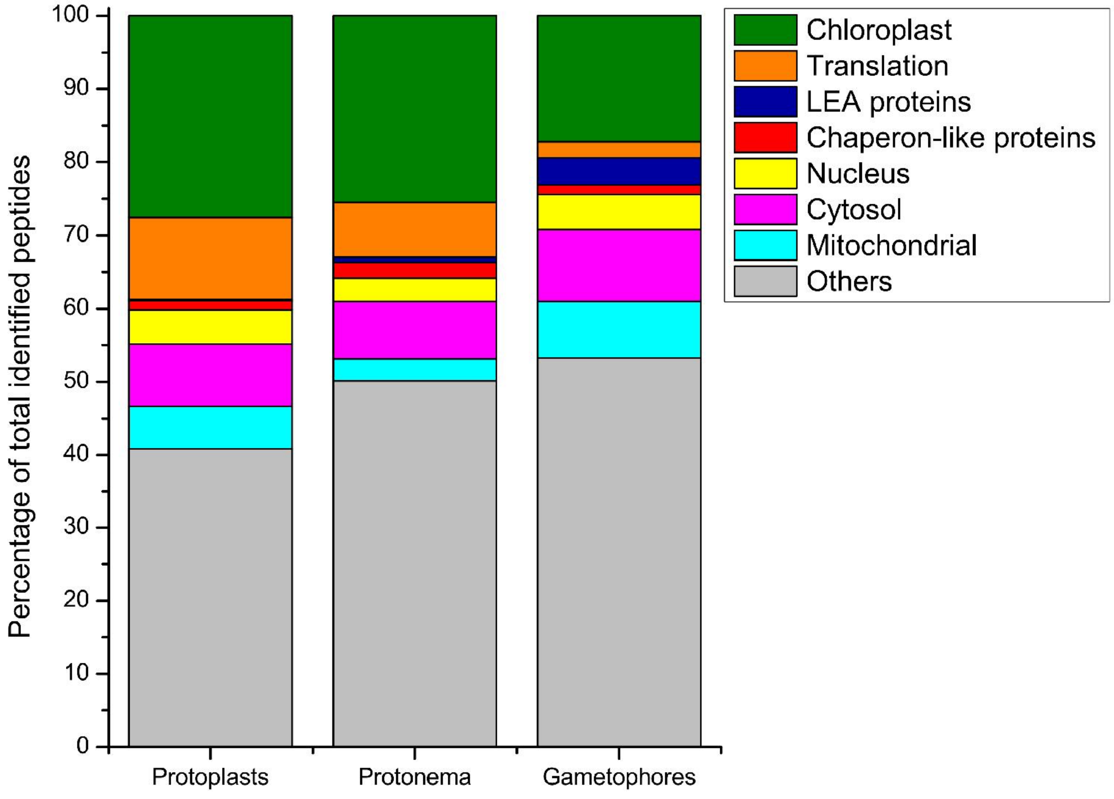

Additional file 6. Ratios of endogenous peptides derived from eight groups of precursor proteins in peptidomes of gametophores, protonemata, and protoplasts. The following groups of proteins were selected for visualization: chloroplast, translation-associated (ribosome proteins and translation elongation factors), mitochondrial, nuclear, LEA and chaperone-like proteins.
