# Supplementary material for: Specific pools of endogenous peptides are present in gametophore, protonema, and protoplast cells of the moss Physcomitrella patens
Source: BMC Plant Biol. 2015 Mar 15;15:87. doi: 10.1186/s12870-015-0468-7 (PMC4365561; doi:10.1186/s12870-015-0468-7)
Supplement: Additional file 9: — Venn diagram showing the amount of peptides identified in protonemata treated with 0.025% (w/v) Driselase compared with untreated protonemata (control). [file 12870_2015_468_MOESM9_ESM.pdf]

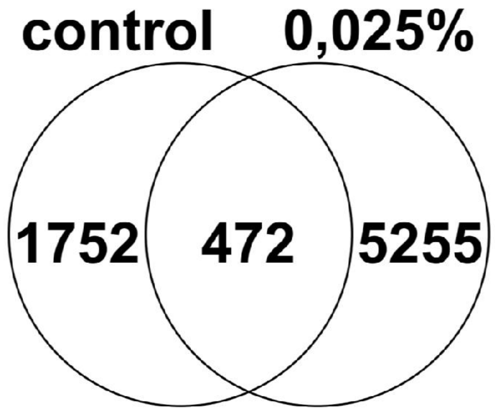

Additional file 9. Venn diagram showing the amount of peptides identified in protonemata treated with 0.025% (w/v) Driselase compared with untreated protonemata (control).
