# Supplementary material for: Specific pools of endogenous peptides are present in gametophore, protonema, and protoplast cells of the moss Physcomitrella patens
Source: BMC Plant Biol. 2015 Mar 15;15:87. doi: 10.1186/s12870-015-0468-7 (PMC4365561; doi:10.1186/s12870-015-0468-7)
Supplement: Additional file 14: — Distribution of differentially expressed (DE) genes over the clusters obtained with the DAVID tools. [file 12870_2015_468_MOESM14_ESM.pdf]

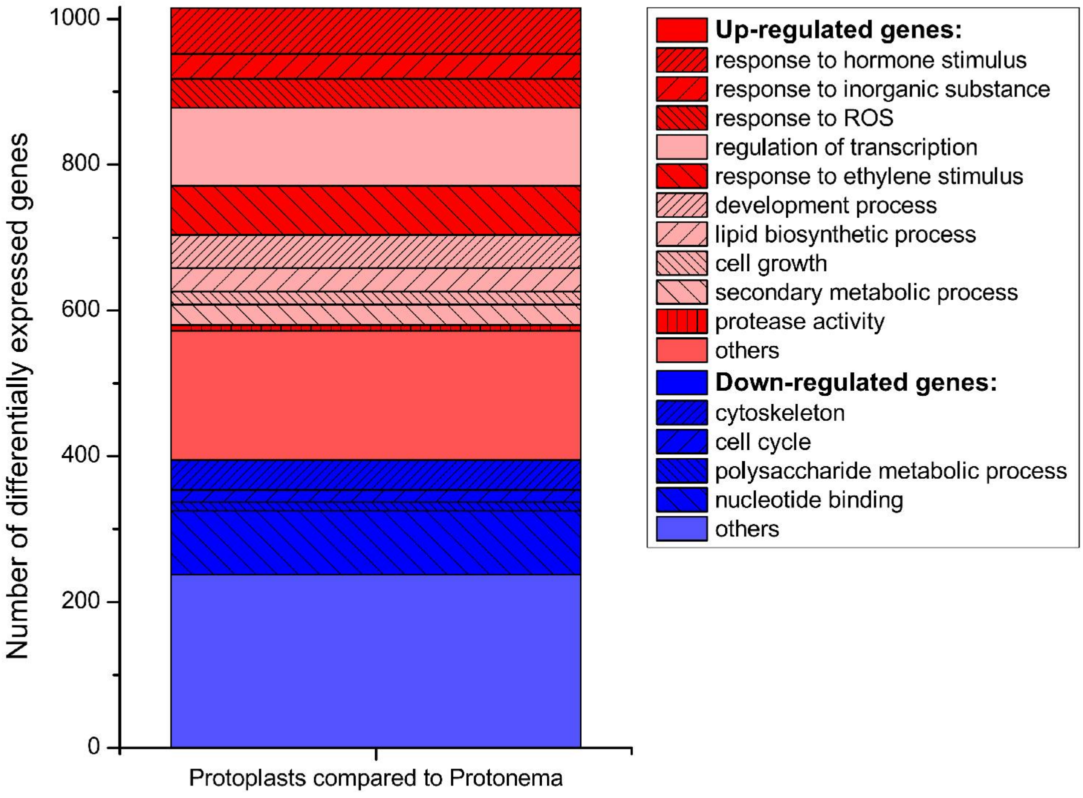

Additional file 14. Distribution of the differentially expressed (DE) genes over the clusters obtained with the DAVID tools.
