# Supplementary material for: Specific pools of endogenous peptides are present in gametophore, protonema, and protoplast cells of the moss Physcomitrella patens
Source: BMC Plant Biol. 2015 Mar 15;15:87. doi: 10.1186/s12870-015-0468-7 (PMC4365561; doi:10.1186/s12870-015-0468-7)
Supplement: Additional file 16: — Change in protein abundance across gametophores and protonemata. [file 12870_2015_468_MOESM16_ESM.pdf]

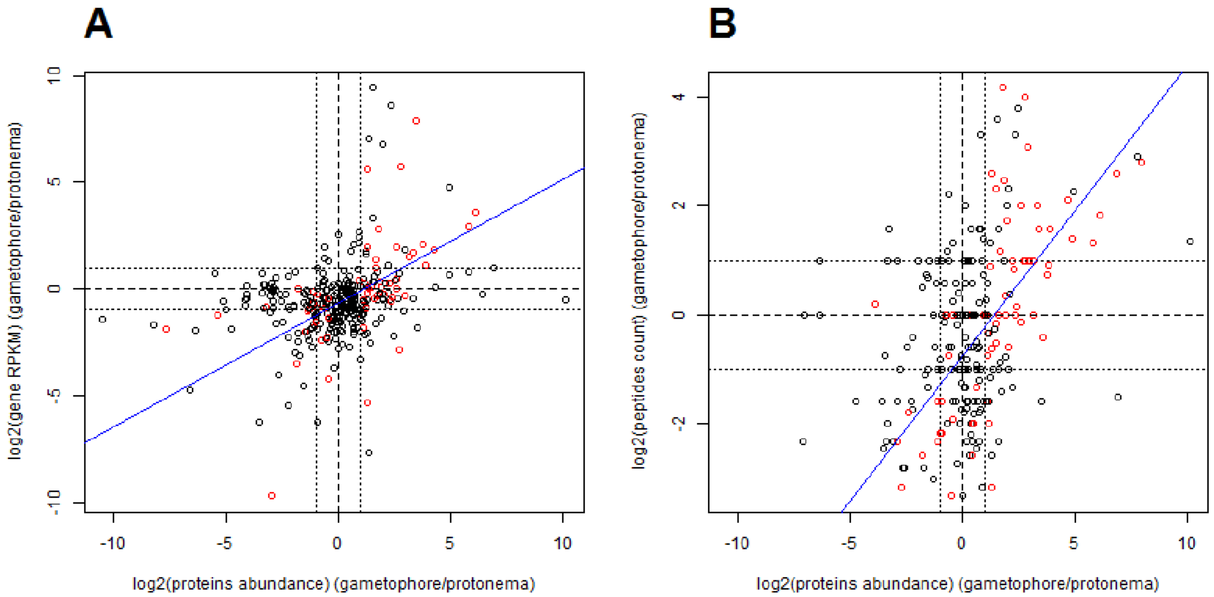

Additional file 16. Change in the protein abundance obtained with the use of a Progenesis LC-MS (Nonlinear Dynamics, Durham NC, USA) software package and gene expression by mRNA-Seq (RPKM) across gametophores and protonemata. (A) Consistency between fold changes in protein and gene abundances for gametophore and protonema. Spearman's correlation coefficient equals 0.6939. **(B)** Consistency between fold changes in protein abundance and the number of identified peptides for gametophore and protonema. Spearman's correlation coefficient equals 0.7371. Reliable changes of protein abundances (p-value < 0.05) between gametophore and protonema are shown as red points.
