# Supplementary material for: Specific pools of endogenous peptides are present in gametophore, protonema, and protoplast cells of the moss Physcomitrella patens
Source: BMC Plant Biol. 2015 Mar 15;15:87. doi: 10.1186/s12870-015-0468-7 (PMC4365561; doi:10.1186/s12870-015-0468-7)
Supplement: Additional file 20: — Time-courses of maximal $\left({\varPhi}_{\max}^{\mathrm{PSII}}\right)$ΦmaxPSII and operating value at moderate light intensity (100 μmol∙m −2 ∙s −1 ; ${\varPhi}_{100\mu E}^{\mathrm{PSII}}$Φ100μEPSII) of quantum efficiency of photosystem II (PSII) photochemical activity in Physcomitrella patens protonema cells under treatment with 0.5%, 0.025%, and 0.0025% Driselase solution. [file 12870_2015_468_MOESM20_ESM.pdf]

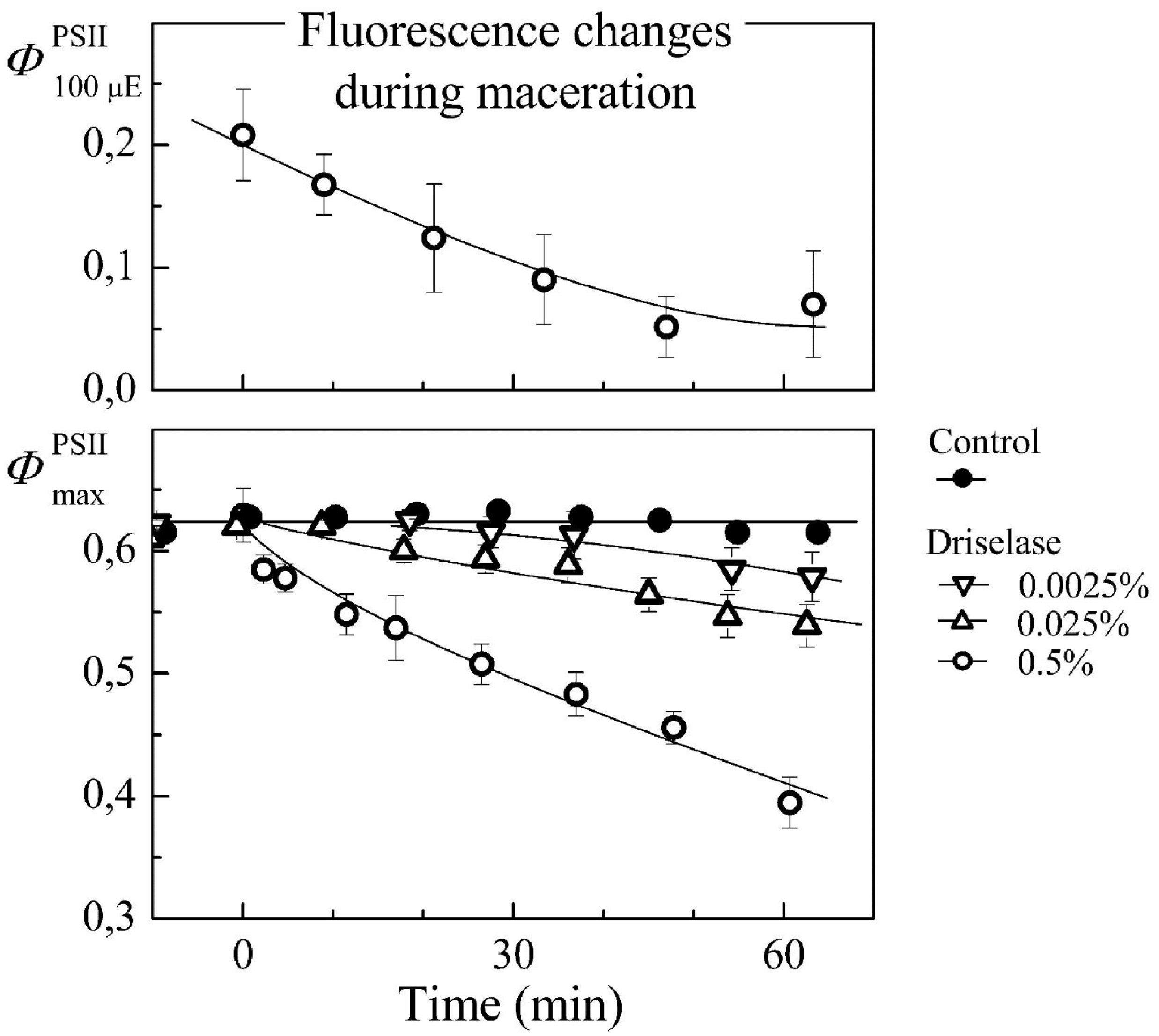

Additional file 20. The time-courses of maximal (Φ^{PSII}_{max}) and operating value at moderate light intensity (100 μmol·m^{-2}·s^{-1}; $\Phi^{PSII}_{100\mu E}$) of quantum efficiency of photosystem II (PSII) photochemical activity in *P. patens* protonema cell under the treatment with 0.5%, 0.025% and 0.0025% driselase solution.
